# Dynamic Nanocrystal-Ligand Boundaries: Reversible Photoinduced Ligand Detachment from Quantum Dots in Solution

**DOI:** 10.1021/jacs.5c19167

**Published:** 2026-01-15

**Authors:** McKenna N. Grega, Jacob A. Cho, Robert A. Brown, John B. Asbury

**Affiliations:** Department of Chemistry, 311285 The Pennsylvania State University, University Park, Pennsylvania 16802, United States

## Abstract

The porosity of ligand shells of colloidal quantum dots (QDs) can influence the overall rate and yield of charge transfer processes occurring at their surfaces. However, the density of ligand shells on QDs can also influence their colloidal and photochemical stability. We used time-resolved infrared spectroscopy to show that photoinduced ligand detachment, the tendency for certain ligands to detach from QD surfaces when the nanocrystals are promoted to their excitonic excited states, can be used to transiently enhance the porosity of oleic acid-passivated CdSe QDs in solution. Furthermore, we synthesized CdSe QDs with varying ligand shell densities to examine the corresponding influence that van der Waals interactions among ligands have on the yield of photoinduced ligand detachment and the time scale on which ligands return to QD surfaces. We observed that oleic acid ligands on CdSe QDs with lower shell densities have a higher probability of escape for longer periods of time. Despite this, oleic acid ligands on fully passivated CdSe QDs are still able to photodetach, resulting in a transient increase of their ligand shell porosity. In contrast, QDs with multilayer ligand coronas exhibit negligible photoinduced ligand detachment because the outer molecular layers introduce a type of cage effect, preventing the escape of the interior ligands. Our findings suggest the intriguing possibility that photoinduced ligand detachment can be used to transiently decrease the density of ligand shells of QDs to facilitate charge transfer processes while still allowing them to be fully passivated between excitation events for photochemical and colloidal stability.

## Graphical abstract

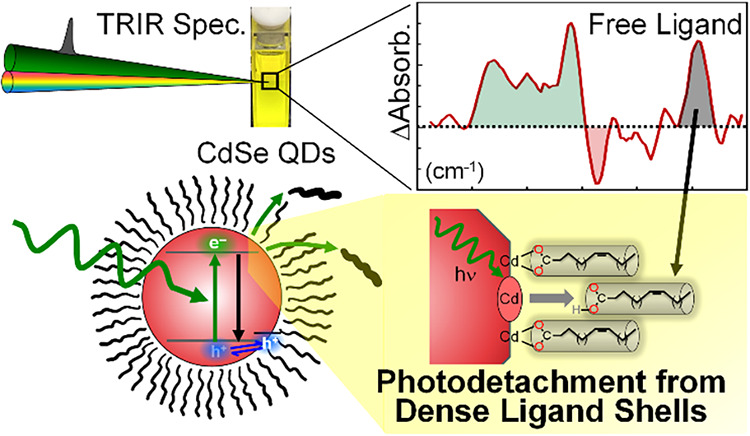

## Introduction

The structure and bonding at the nanocrystal-ligand boundary of colloidal quantum dots (QDs) determine the energetic barrier heights and widths through which energy and charge transfer processes occur at their surfaces.^1–17^ These barriers influence the balance of forward versus back electron transfer rates that can modulate the overall quantum yield of photocatalytic transformations^18–32^ driven by excited QDs in solution.^1,5,8–14^ For example, transitions between ordered and disordered phases of alkyl thiol ligands on PbS modulate the formation of surface trapped electrons^15^ and influence the ability of molecular species like viologens to approach QD surfaces to undergo charge transfer within the excited state lifetime of the nanocrystals.^16^ The interplay of forward versus back charge transfer processes is also influenced by the molecular motion of charge acceptors at QD surfaces,^17^ which in turn is influenced by the permeability of their ligand shells.^1^

The molecular dynamics of ligands are also often coupled to charge dynamics at QD surfaces,^33–36^ which can influence their photocatalytic activity.^17,37–39^ For example, the transfer of localized holes to sacrificial ligand donors such as mercaptoproprionic acid extends the charge separated lifetime of CdSe-Pt photocatalysts and enhances the efficiency of the hydrogen evolution reaction.^17,37,38^ In other examples, change transfer across QD surfaces involves surface traps,^39,40^ while trapped electrons most likely at surfaces can modulate the emission characteristics of QDs by enhancing Auger relaxation, a process known as quantum dot “blinking”.^41–43^ Furthermore, recent time-resolved infrared investigations of the excited state surface chemistry of QDs revealed that ligands can transiently photodetach from their surfaces, temporarily creating a more permeable ligand shell^33–35^ – a process that we will show below results from interactions of ligands with surface electron density.^36^

The intriguing idea that photoinduced ligand detachment might be used to transiently create more permeable ligand shells suggests the need to investigate the role that van der Waals interactions among ligands may have in mediating this process. For example, studies of photocatalytic production of H_2_ in solution revealed a slow degradation of catalytic performance that could be recovered by subsequent addition of the original ligands to the solution,^6^ revealing that the diffusional encounter of free ligands with QDs could restore their ligand shells.^44^ Therefore, we explored the influence that the density of van der Waals interactions might have on ligand photoinduced detachment kinetics by varying the porosity of the ligand shells on CdSe QD surfaces. We first examined the excited state surface chemistry of CdSe QDs with moderate ligand shell densities to establish that photoinduced ligand detachment does in fact occur in solution as was observed in dry films.^35^

We hypothesized that molecular friction arising from van der Waals interactions among adjacent ligands may inhibit photoinduced ligand detachment in dense ligand shells and reduce the potential to transiently create more permeable ligand shells in the excited states of QDs. To test this hypothesis, we used varying amounts of oleic acid (OA) in the synthesis of CdSe QDs^45^ to adjust their ligand shell densities^46,47^ while keeping all other properties the same including their bandgaps, nanocrystal sizes, and photoluminescence (PL) spectra. We photoexcited the CdSe QDs in solution and directly measured the transient vibrational spectra of their photodetached ligands, which allowed us to track the fraction of ligands that successfully escaped their surfaces. We correlated this fraction of ligands that escaped with the density of the ligand shells determined by NMR spectroscopy.^46,48,49^ We reveal that ligands attached to QDs with lower density ligand shells have a higher probability of escape from their shells for longer periods of time. Furthermore, we show that ligands on fully passivated QDs are still able to photodetach and reattach to the QD surfaces on the microsecond time scale. In contrast, CdSe QDs with excessive multilayer ligand shells exhibit the reduction in photodetachment yields that we hypothesized would occur because the outer ligand layers impede the motion of ligands attached to their surfaces. While these observations were made in an inert solvent and N_2_ saturated environment, a logical inference of our findings is that it may be possible to use this type of transient increase of ligand shell porosity in the excited states of QDs. This may enhance the ability of QDs to drive charge transfer processes while still allowing them to be fully passivated for photochemical and colloidal stability between excitation events. Future work exploring the influence that dynamics of the nanocrystal-ligand boundary have on photocatalytic systems will be of significant interest.

## Experimental Methods

The synthesis of CdSe QDs was adapted from a procedure outlined by Cogan et al.^45^ For ∼ 3.0 nm QDs, 0.1711g CdO, 3.33 mL 1-octadecene (ODE), and variable amounts X mL of OA (X = 1.0 mL (3:1), 1.5 mL (4.5:1), 2.0 mL (6:1), or 2.5 mL (7.5:1)) were heated to 120 °C under vacuum to degas the precursors. Once the temperature reached 120 °C, the reaction flask was kept under N_2_ flow for the remainder of the nucleation process. In parallel, 0.227g elemental Se was added to 3.33 mL ODE and sonicated for 10 min until a Se suspension formed. The reaction flask was heated to 260 °C causing the solution to become yellow and clear. Then, 0.5 mL of the Se-ODE suspension was injected, and the reaction flask was kept at 260 °C for 1 min. Following the 1 min reaction time, the reaction flask was removed from heat and allowed to cool to 200 °C before using a water bath to cool the flask to room temperature. Once the reaction flask cooled, the red product was evenly distributed among three test tubes, and 200 proof ethanol was added in a 6:1 ethanol to reaction mixture ratio. The three test tubes were then subjected to centrifugation at 4000 rpm for 10 min, and the supernatant containing excess oleic acid and ODE was discarded. After dispersion in minimal hexanes, the product was washed once more using the same procedure, and the collected precipitate was redispersed in CCl_4_.

Infrared spectroscopy measurements of CdSe QDs in solution were performed using a 150 μm path length in a demountable Harrick liquid cell with CaF_2_ windows. The solutions were sparged with nitrogen for 15 min prior to loading into the Harrick cell to minimize the exposure of the QDs to oxygen.

Mid-IR transient absorption experiments were performed using a table top transient absorption instrument, which consisted of a pulsed, frequency doubled (532 nm) Nd:YAG laser operating at 5 kHz as the excitation source and a SiC glowbar as the probe light source. The probe light source was dispersed in a monochromator and detected with a cryogenically cooled mercury cadmium telluride (MCT) photovoltaic detector.

Visible absorption measurements were performed with a Beckman DU 720 UV/vis spectrometer. The spectra were background subtracted with the appropriate solvent in a 1 cm path length quartz cuvette. Steady-state PL spectra were measured using a home-built spectrometer with a frequency tripled (355 nm) Nd:YAG laser operating at 30 Hz. A photodiode optimized for detection in the visible and near-IR region was used as the detector, and the time-dependent response of the photodiode was integrated to produce the steady-state PL spectra. As mentioned above, all liquid samples used in these studies were sparged with nitrogen for 15 min prior to the measurements. FTIR spectra were measured using a commercially available instrument (JASCO, 6600 FTIR Spectrometer equipped with a liquid nitrogen cooled MCT detector).

Colloidal CdSe nanocrystal solutions with native oleate ligands were drop-cast onto carbon grids and high resolution, scanning transmission electron microscopy (STEM) and transmission electron microscopy (TEM) were performed. The STEM and TEM images were collected on a FEI, Talos F200X with an XFEG source at 200 kV and were processed using ImageJ software.

NMR samples were prepared by adding ∼100 mg of dried (3:1, 4.5:1, 6:1, 7.5:1) CdSe directly to NMR tubes containing 600 μL toluene-d_8_ with ∼5 mg of ferrocene as an internal standard. All NMR measurements were done using a Bruker AVANCE NanoBay NEO-400 spectrometer. Following spectra collection, peak integration was done via TopSpin software.

## Results and Discussion

### Photoinduced Ligand Detachment from CdSe QDs in Solution

We investigated the photoinduced ligand detachment kinetics of OA passivated CdSe QDs in solution using time-resolved infrared (TRIR) spectroscopy (Figure 1A). We sought to determine whether it is possible to transiently enhance the porosity of their ligand shells in the liquid environment. To explore this, we synthesized CdSe QDs following literature procedures^45^ and used a concentration of OA in the reaction flask that was sufficient to achieve a 3.0:1 mol ratio between OA and Cd precursors during the synthesis. We will refer to QDs synthesized under these conditions as 3.0:1 CdSe QDs here and going forward. This concentration of OA produced colloidally stable, monodisperse CdSe QDs of approximately 3.0 nm diameter as indicated by their absorption and photoluminescence spectra represented in Figure 1B.^50,51^ A scanning transmission electron microscopy (STEM) image of the QDs appears as the inset, further confirming the approximate size and monodispersity of the nanocrystals.

**Figure 1:**
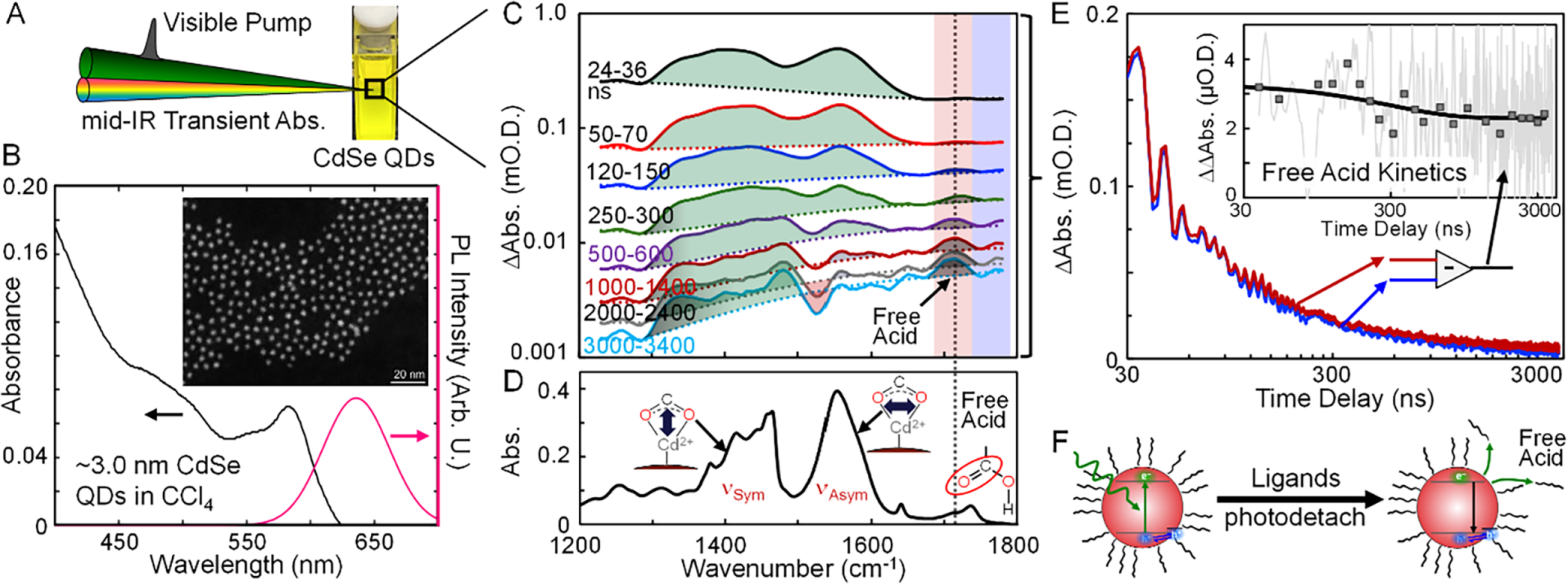
(A) Time-resolved infrared spectroscopy was used to investigate photoinduced ligand detachment from CdSe QDs in solution. (B) Visible absorption and PL spectra of 3.0:1 CdSe QDs in CCl_4_. The inset represents a STEM image of the CdSe QDs. (C) Time-resolved infrared spectra at a range of time delays of a solution of 3.0:1 CdSe QDs in CCl_4_ following excitation at 532 nm. The transient spectra are plotted on a logarithmic amplitude scale to represent their amplitudes over their full time-evolution. The spectra are time-averaged over time periods indicated in the legend. Broad 1S-1P intraband transitions in each spectrum are indicated by the dotted lines. Fano resonance features are shaded in green, while the C =O stretch of photodetached free acid ligands is shaded in gray. The red shading highlights where kinetics of the free acid peak were measured. The blue shading marks where the kinetics of the 1S-1P transition were recorded. (D) The FTIR spectrum of the sample aids in the vibrational peak assignments. (E) Transient absorption kinetics traces measured at the frequency of the free acid peak (red trace) and 1S-1P transition (blue trace). The difference of these traces isolates the time dependence of the free acid peak and is represented as the gray data in the inset. The primary Free Acid Kinetics data were time averaged over intervals represented by the square symbols to highlight the prompt formation and persistent lifetime of the photodetached ligands in solution. (F) Cartoon illustration of ligand photodetachment from CdSe QDs.

Figure 1C presents TRIR spectra measured of the 3.0:1 CdSe QDs dispersed in CCl_4_ solution in the region of the carboxylate vibrational features of OA ligands that were bonded to the surfaces of the nanocrystals.^52,53^ The concentration of QDs in the CCl_4_ solution used in the experiment was selected to yield the ground state infrared absorption spectrum depicted in Figure 1D when a 150 μm path length was used. In the TRIR experiment, the primary spectra were recorded every 2 ns following optical excitation at 532 nm near the excitonic bandgap of the QDs. To enhance their signal-to-noise ratio, the TRIR spectra appearing in Figure 1C represent averages over multiple time points at each frequency. Spectra plotted on the earliest time delay were averaged over 24–36 ns. Spectra plotted on longer time scales were averaged over larger time intervals as represented in the legend.^35^ This processing step permitted the spectra to be represented on a logarithmic amplitude scale while still preserving sufficient time-resolution to resolve the transient vibrational features of ligands that were perturbed by optical excitation of the QDs. The TRIR spectra time-averaged over the same intervals appear in Figure S1A on a linear amplitude scale for reference.

The TRIR spectra exhibit three types of features that are characteristic of prior TRIR measurements of CdSe QDs of this nanocrystal size and in this spectral region.^33–35^ First, a broad electronic absorption feature appears as a time-dependent offset in the TRIR spectra measured at each time delay.^54^ These offsets are indicated by the dotted lines under each spectrum. We intentionally measured a broader range of frequencies around the carboxylate vibrational features of the OA ligands so that the broad absorption offsets could be measured and fit with third order polynomial functions as we have done in our prior work.^33–35^ This electronic absorption feature is consistent with prior measurements^55–58^ and results from the 1S-1P intraband transition of the CdSe QDs that were optically excited to their excitonic states. The larger size of the CdSe QDs examined here in comparison to our prior studies^35^ caused the 1S-1P transition of electrons to appear in the region of the carboxylate stretch vibrational modes of the surface attached ligands.

The second type of features in the TRIR spectra are highlighted in green in Figure 1C. These are Fano resonances^59^ resulting from coupling of the vibrational transitions of the carboxylate anchoring groups of ligands with the 1S-1P transitions of the QDs that occur in the same frequency range.^60^ The Fano resonances correspond to the symmetric and antisymmetric carboxylate stretch modes of the ligands. This assignment is supported by the similarity of the Fano resonance features with the FTIR spectrum of the sample showing the ground state vibrational features of the ligands, which are specific to carboxylate groups bonded to Cd atoms on the surfaces of the CdSe QDs.^52,53^ We tested the Fano resonance assignment of the green-shaded features by comparing their time-dependence to that of the 1S-1P transition of the QDs. We reasoned that the Fano resonances should decay synchronously with the 1S-1P transition if they arise from coupling of the ligand vibrational modes with the electronic transition. Figure S2A represents the same TRIR spectra that appear in Figure 1C and includes yellow shading at the 1300 and 1670 cm^–1^ frequencies at which the time-dependence of the 1S-1P transition was measured. The corresponding kinetic decay trace of the 1S-1P transition appears in Figure S2B with an inset illustrating the electronic transitions giving rise to the broad absorption in the TRIR spectra.

Figure S2A also highlights with green shading the 1480 cm^–1^ frequency where the Fano resonance kinetics were measured. Kinetics measured at this frequency contain contributions both from the 1S-1P electronic absorption and the Fano resonance. To isolate the Fano resonance kinetics specifically, we subtracted the kinetics of the 1S-1P absorption (yellow trace in Figure S2B) from the kinetics measured at 1480 cm^–1^. The difference resulting from this subtraction appears as the green trace in Figure S2B and represents the time-dependence of the Fano resonance amplitude from the sample. The comparison of the 1S-1P (yellow) and the Fano resonance (green) kinetics demonstrates that they decay synchronously, which supports the assignment of the carboxylate vibrational features of ligands attached to the CdSe QDs being Fano-type resonances. The best fit curve (dotted line) through the kinetics traces indicates the average lifetime of the 1S-1P and Fano resonance features was 180 ± 30 ns with best fit parameters tabulated in Table S1. The error range in the average lifetime is dominated by uncertainty in the amplitude of the longest decay component of the triexponential fit function.

It is interesting to note that the Fano resonance features observed in the CdSe QDs in CCl_4_ are qualitatively different in shape from our prior measurements of similar carboxylate features of ligands attached to QD surfaces in dry films (no solvent).^33–35^ In dry films, the transient ligand vibrational features exhibited positive and negative-going absorptive line shapes that corresponded to the FTIR spectra of the samples. We believe that the difference in the shapes of the Fano features measured here versus in dry films arises from the ability of ligands to partition between being attached to QD surfaces versus being dispersed in the surrounding liquid (this work). This leads to QDs having undercoordinated Cd atoms at their surfaces that can facilitate electron localization by contributing midgap states near the lowest unoccupied molecular orbitals of the QDs.^61–66^ The shapes of Fano resonance features are known to depend sensitively on the coupling of the electronic and vibronic coordinates.^59^ Therefore, the localization of surface electron density at undercoordinated Cd atoms of QDs in solution enhances the coupling of vibrational transition dipole moments of nearby ligands to the electronic transitions of the QDs. This changes the Fano resonance shapes to being all-positive in the 3.0:1 CdSe QDs examined here. The localization of surface electron density at undercoordinated Cd sites can also increase the density of charge recombination centers that lead to faster excited state decay.^61,67–69^ As we will show below, increasing the density of ligands on QDs in solution changes the shapes of their Fano resonances to more closely resemble those measured in film environments.^35^ The excited state lifetimes of the QDs with denser ligand shells also increase, which is consistent with reduction of undercoordinated Cd atoms, decrease of localized surface electron density, and fewer recombination centers.

The third type of feature in the TRIR spectra in Figure 1C appears around 1720 cm^–1^ and has a symmetric absorptive-type line shape. This feature is highlighted in gray shading and is apparent in the TRIR spectra measured on the hundred nanosecond to microsecond time scales when they are plotted on a logarithmic amplitude scale. This feature is assigned to the C=O vibrational stretch mode of protonated carboxylic acid groups of OA ligands that photodetached from CdSe QD surfaces following optical excitation. The assignment of this feature specifically to protonated ligands that photodetached from QD surfaces is based on its similarity to the free acid C =O peak at 1720 cm^–1^ with similar line shape in the FTIR spectrum of a solution consisting of neat OA dissolved in CCl_4_ in Figure S3. We label this 1720 cm^–1^ absorptive peak the “free acid” feature in the TRIR spectra in Figure 1C.

We tested the assignment of the 1720 cm^–1^ peak to free acid groups that photodetached from QDs by comparing its time-dependence to the kinetics of the 1S-1P electronic transition. We hypothesized that the free acid peak should not follow the same time-dependence as the kinetics of the 1S-1P electronic transition because the free acid groups are no longer attached to the QD surfaces and are therefore no longer coupled to their electronic transitions. To make the comparison, we extracted the kinetics of the 1S-1P transition at 1770 cm^–1^ (blue shaded vertical line in Figure 1C) and overlaid them with the kinetics measured at 1720 cm^–1^ at the peak of the free acid feature, indicated by red shading. The corresponding kinetics traces with matching color coding appear in Figure 1E. The difference of these kinetics traces represents the time-dependence of the free acid peak itself, which appears in the inset in Figure 1E with the label “Free Acid Kinetics”.

We recognize that the free acid transient absorption signal at 1720 cm^–1^ is relatively small and has a finite signal-to-noise ratio. Therefore, we employed the same type of time-averaging approach used to present the TRIR spectra in Figure 1C to reduce the noise in the free acid kinetics trace. The resulting down-sampled free acid kinetics data appear as the square symbols in the inset of Figure 1E. The square symbols were processed by averaging the primary free acid kinetics data (measured every 2 ns) over variable time ranges. For example, the first three square symbols at earlier time delays represent averages over 24–36, 38–50, and 52–72 ns, respectively. While, the last three square symbols at longer time delays represent averages over 2660–2960, 3000–3300, and 3330–3630 ns, respectively. The complete set of time-averaging windows used to create the down-sampled kinetics data are provided in Table S2. The time ranges over which the down-sampled kinetics data were averaged are described by the approximate widths of the symbols in the inset on the logarithmic time axis, showing that the averaging approach preserves the time-resolution of the data that is needed to follow the time-dependence of the free acid peak. The smooth curve through the square symbols is a guide to the eye showing that the free acid peak appears in the spectra at the earliest times measured (24–36 ns) and retains approximately 70% of its initial amplitude of 3 ± 0.3 μO.D. into the microsecond time scale within experimental precision. This time-dependence does not match the kinetics of the 1S-1P transition, indicating that the 1720 cm^–1^ peak is not a Fano-type resonance.

Finally, we confirmed by independent analysis that the free acid peak appears promptly after photoexcitation and retains most of its amplitude into the microsecond time scale by examining the transient vibrational spectra of the 3.0:1 CdSe QDs on a linear amplitude scale in Figure 2. To do this, we fit the broad 1S-1P electronic absorptions at each time delay using a third-order polynomial fit function (dotted curves in Figure 1C) and subtracted them from the corresponding TRIR spectra to obtain the transient vibrational spectra represented in Figure 2. The data reveal the appearance of a few μO.D. signal at 1720 cm^–1^ in the earliest-time spectrum (labeled Free Acid) that retains most of its initial amplitude throughout the microsecond time scale, consistent with the free acid kinetics trace in Figure 1E. We note that the free acid peak is not visible in the earlier-time TRIR spectra in Figure 1C because the other features in the spectra have much larger amplitudes. It is only after the initial Fano resonance and 1S-1P electronic absorption features decay on longer time scales that the smaller free acid feature becomes apparent when plotted on a logarithmic amplitude scale.

**Figure 2:**
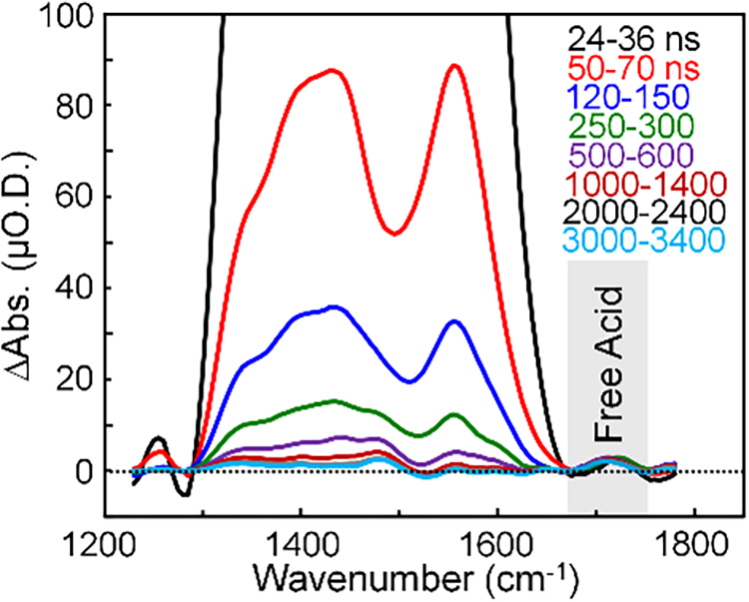
Transient vibrational spectra of a solution of 3.0:1 CdSe QDs in CCl_4_ following excitation at 532 nm. The data are obtained by subtraction of the broad 1S-1P electronic transitions from the data in Figure 1C. The subtracted spectra are plotted on a linear scale and represented on a smaller amplitude window to show that the free acid peak at 1720 cm^–1^ of photodetached ligands appears within the earliest time delay measured and persists throughout the microsecond time scale.

We analyzed the amplitude of the free acid peak in the TRIR spectra to estimate the quantum yield of photoinduced ligand detachment per absorbed photon. We could do this because its amplitude is not enhanced by coupling to the 1S-1P electronic transition, which is confirmed by its unique kinetics with respect to the 1S-1P transition. Therefore, we could quantitatively interpret the change in absorption of this peak in terms of the change in concentration of free ligands by independently measuring the absorption coefficient of the free acid peak, the path length of the sample, and the absorbed excitation density from the optical pump pulse used in the TRIR experiment. The FTIR spectrum in Figure S3 was obtained from a solution containing 20 mM OA in CCl_4_ and measured with a 150 μm path length between two CaF_2_ windows. The absorbance of 0.3 of the free acid peak indicates it has a molar absorption coefficient of 1000 M^–1^ cm^–1^ within experimental precision. The excitation intensity (20 μJ/cm^2^ per pulse), absorbance of the sample at 532 nm of 0.1, free acid transient absorption amplitude of 3 ± 0.3 μO.D., and the beam overlap factor of the TRIR instrument led us to compute a quantum yield of 70% ± 10% for ligand photodetachment per absorbed photon as described in Note S1. The uncertainty in this quantum yield is dominated by the finite signal-to-noise of the free acid peak as indicated in Figure 1E, which resulted in the experimental uncertainty of 3 ± 0.3 μO.D. as mentioned above. Because the excitation intensity was selected to excite no more than one out of every ten CdSe QDs in the sample per pulse, no more than 1% of the CdSe QDs absorbed two photons per pulse in the TRIR experiment. The approximately 70% quantum yield for photoinduced ligand detachment is significantly larger than this value, indicating that the photodetachment process is dominated by single-photon absorption events.^70^

It is instructive to consider the mechanistic origin of the photoinduced ligand detachment process from the CdSe QDs. Prior work examining the interaction of X-type ligands on QD surfaces revealed that the introduction of electrons into QDs by chemical reduction led to the loss of carboxylate ligands.^36^ Our observation of all-positive Fano resonances in 3.0:1 CdSe QDs indicates that carboxylate ligands interact strongly with localized surface electron density that forms at undercoordinated Cd sites in the excited states of the QDs. The observation that 3.0:1 CdSe QDs have shorter excited state lifetimes than were measured in dry films^35^ or in CdSe QDs with more highly coordinated surfaces (see below) is again consistent with undercoordinated Cd atoms leading to the localization of surface electron density and serving as charge recombination centers. These observations lead us to conclude that it is the localization of surface electron density in the excited states of QDs that causes photoinduced ligand detachment from their surfaces. This mechanism of ligand photodetachment is essentially an excited state version of the process identified in ref 36. This conclusion is further supported by the ligand density dependent study described below in which we varied the density of undercoordinated Cd atoms on QD surfaces and observed a reduction of the ligand photodetachment quantum yield.

We note that the solution environment appears to facilitate the protonation of the carboxylate groups of OA ligands that photodetached from CdSe QD surfaces. It is likely that weekly bound oleic acid ligands that were protonated and near the photodetachment sites provided the source of protons that led to protonation of the photodetached ligands.^46^ This protonation following ligand photodetachment, which was not observed in our prior work on dry CdSe QD films,^35^ allows us to track the transient free ligand concentration and observe their long-lived dissociation from nanocrystal surfaces. This protonation also indicates that the OA ligands did not photodetach from the QD surfaces still bonded to the Cd atoms by which they were anchored to their surfaces. Instead, the ligands appear to detach as free carboxylates that then become protonated before the first TRIR spectrum was recorded on the 24–36 ns time scale following photoexcitation.

Finally, Figure 3 reveals that the photodetachment process is fully reversible in the 3.0:1 CdSe QDs in solution. This is demonstrated by comparing FTIR spectra of the sample measured before versus after prolonged excitation at 532 nm under conditions exactly matched to the TRIR measurements. Each CdSe QD in the sample underwent on average ∼10^7^ excitation events over the course of the 4 h exposure, with each absorbed photon having a quantum yield of about 70% to photodetach a ligand. However, the FTIR spectrum of the sample was unchanged within experimental precision after these excitation events – indicating reversibility.

**Figure 3:**
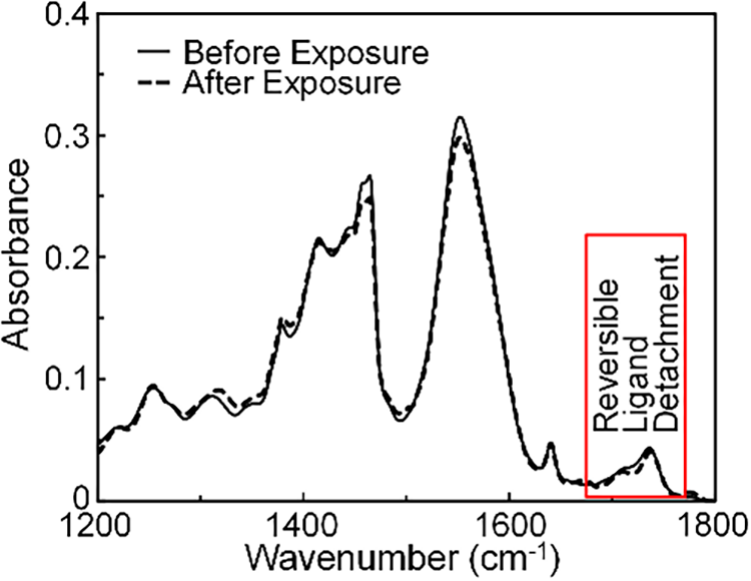
Comparison of FTIR spectra of a solution of 3.0:1 CdSe QDs in CCl_4_ that were measured before versus after exposure of the sample to 20 μJ/cm^2^ 532 nm excitation with 5 kHz repetition rate over a 4 h period. Under these conditions, each QD in the solution absorbed ∼10^7^ photons on average. The spectra show the reversibility of the photoinduced ligand detachment within experimental precision.

### Impact of Interactions among Ligands on Photodetachment

The free acid kinetics trace in the inset of Figure 1E reveals that ligands photodetach from CdSe QD surfaces in solution and remain detached on the microsecond time scale, indicating that the ligands diffuse away from the nanocrystal surfaces for extended periods of time. This suggests that van der Waals interactions among ligands may figure prominently in mediating the rate and yield of ligand photodetachment and can impact the design of ligand shells that are capable of transiently increasing their porosity following photoexcitation. Therefore, we undertook a study of the influence that ligand shell density of OA-passivated CdSe QDs has on the yield of photoinduced ligand escape and the rate of their return to QD surfaces in an effort to elucidate the influence that interactions among ligands have on this process.

To vary the ligand shell density of CdSe QDs examined in this study, we modified the synthesis procedures^45^ by inclusion of increasing concentrations of OA ligands to the reaction flask followed by purification. These concentrations were set to achieve OA:Cd molar ratios of 3.0:1, 4.5:1, 6.0:1, and 7.5:1 in the precursor solutions, respectively. All other reaction conditions were held constant among the synthetic trials. Each reaction condition was repeated a minimum of three times, and the resulting CdSe QDs were purified and analyzed using NMR spectroscopy to quantify the density of OA ligands attached to their surfaces.^46,48,49^ The CdSe QDs were also examined using visible absorption, photoluminescence, and FTIR spectroscopy to compare the variation among their polydispersity, optical bandgaps and ligand surface chemistries as the ligand densities were systematically changed. Here and going forward, we will refer to the CdSe QDs examined in this study by the OA:Cd ratios that were used in their synthesis.

Figure S4 shows representative visible absorption and photoluminescence spectra of the 3.0:1, 4.5:1, 6.0:1, and 7.5:1 CdSe QDs in CCl_4_ examined in this study. Inspection of the absorption and photoluminescence spectra reveals that all CdSe QDs had similar excitonic absorption peaks around 580 nm and photoluminescence peaks around 640 nm. This indicates that the QDs synthesized with different OA:Cd molar ratios had similar sizes and polydispersity. Comparison of the optical bandgaps with published optical gap/diameter correlations confirms that the CdSe QDs were 3.0 nm in diameter.^71^ Figure S5 depicts FTIR absorption spectra of the 3.0:1, 4.5:1, 6.0:1, and 7.5:1 CdSe QDs in CCl_4_. The spectra include characteristic symmetric and antisymmetric stretch modes of the carboxylate anchoring groups of the ligands around 1450 and 1550 cm^–1^, respectively. The 1550 cm^–1^ peak includes some contribution from an overtone transition of the CCl_4_ solvent as well. A small free carboxylic acid peak is also visible around 1720 cm^–1^ in the samples because ligands can partition between surface bonded and solution environments.

Figure 4A presents a ^1^H NMR spectrum of 7.5:1 CdSe QDs dispersed in CCl_4_ with ferrocene added as an internal standard to quantitatively assess the concentration of OA ligands that were free in solution versus those that were attached to the QD surfaces.^46,48,49^ Following the analysis described in refs,^46,48,49^ the vinyl protons of OA in the 5.4–6.0 ppm range exhibit unique peak widths that indicate whether the OA molecules are confined to the vicinity of the QD surfaces or whether they are dispersed and free to diffuse in solution, as indicated by the inset. We computed the concentration of OA ligands confined to the QD surfaces by integrating the “Confined to QDs” OA peaks in each NMR spectrum of the samples. These areas were compared to the integrated area of the ^1^H resonance at 4.0 ppm of the ferrocene internal standard (Fc) to quantify the confined OA concentration for each sample. The original NMR spectrum of each sample in triplicate with insets focused on the 5.4–6.0 ppm region of the vinyl protons of OA appear in Figures S6–S9.

**Figure 4:**
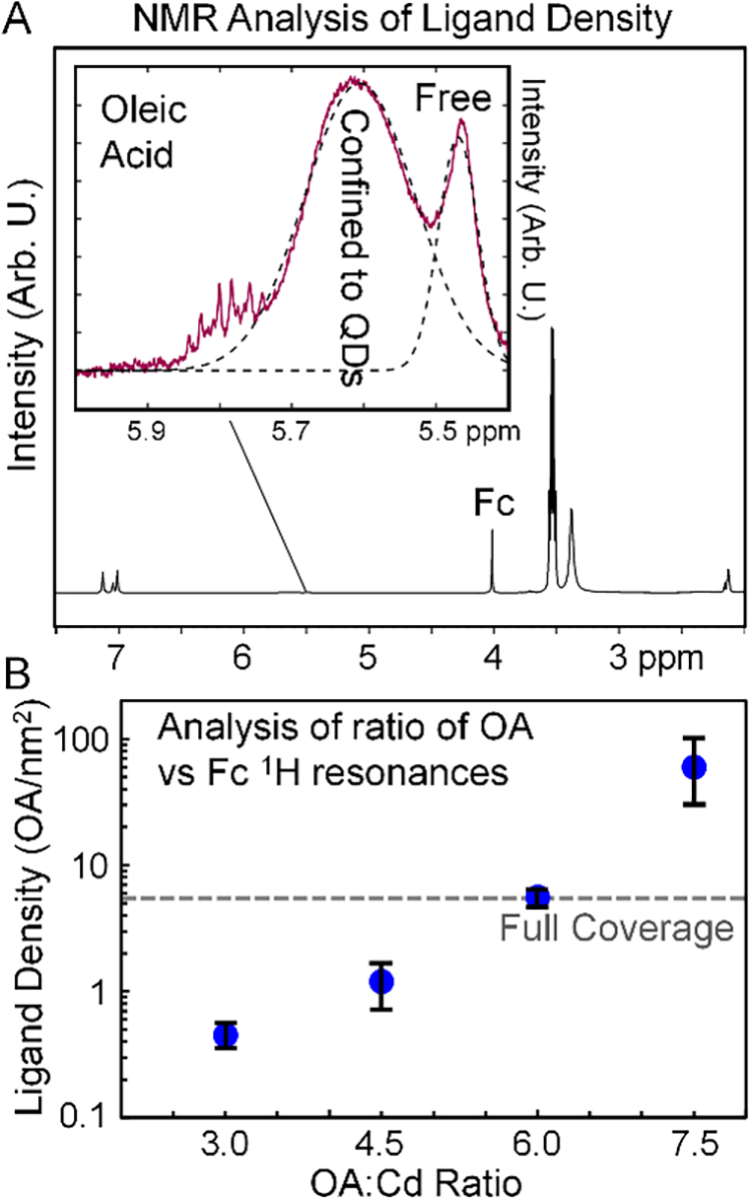
(A) NMR spectrum of a solution of 7.5:1 CdSe QDs in toluene-d8. The inset focuses on the vinyl protons of oleic acid that exhibit distinct line shapes when they are confined to the vicinity of the CdSe QD surfaces versus when they are free to diffuse in solution. (B) Analysis of the Confined vinyl proton peak of oleic acid ligands in the NMR spectra. Comparison of the integrated peak intensity of the confined vinyl proton signal with the integrated peak intensity of ferrocene protons permitted quantitation of the ligand density in each sample. The line labeled “Full Coverage” indicates the approximate density of ligands sufficient to bond to every available Cd atom on the surface of a [100] facet of CdSe.

The concentrations of the confined OA ligands were compared to the concentrations of the CdSe QDs as determined by the excitonic absorption amplitudes of the samples^71^ appearing in Figure S4 to obtain an average number of ligands per QD in the 3.0:1, 4.5:1, 6.0:1, and 7.5:1 CdSe samples as described in Note S2. Then, the surface densities of the OA ligands on the QDs were estimated by accounting for the average surface area of the QDs from their 1.5 nm radii estimated from the wavelength of their excitonic peaks in Figure S4.^71^ Figure 4B displays the estimated ligand densities per nm^2^ on a logarithmic scale of the CdSe QDs obtained from this analysis method. These values are also tabulated in Table S3. The symbols in the figure indicate the average ligand densities of the QDs from the multiple syntheses and measurements, while the error bars indicated the upper and lower bounds of the spread from multiple measurements for each OA:Cd ratio.

The data reveal that the 3.0:1 and 4.5:1 CdSe QDs have ligand densities around 1 OA/nm^2^. Presumably, other surface sites of these QDs were coordinated by other molecular species such as residual Se, the vinyl groups of ODE, or by solvent molecules. Prior studies also revealed differing densities of ligands attached to various crystal facets of nanocrystals.^47^ We note that the 6.0:1 CdSe QD sample exhibits significantly higher ligand density around 5 OA/nm^2^ in comparison to the lower ligand concentrations. The surface density of Cd atoms on a [100] facet of CdSe can be estimated from its lattice constant, a ≅ 4.3 Å, which leads to a Cd surface atom density around 6/nm^2^. Therefore, we conclude that the 6.0:1 CdSe QDs surfaces were fully coordinated by OA ligands within experimental precision. Furthermore, the 7.5:1 CdSe QD sample exhibits dramatically higher ligand shell densities computed to be around 60 OA/nm^2^ from the NMR analysis. The similarity of the visible absorption, PL, and FTIR spectra of the 7.5:1 CdSe QD samples to the others with lower ligand densities indicates that the inorganic cores in all samples had the same size and concentration. Therefore, this extreme ligand density suggests that excess OA ligands in the 7.5:1 CdSe sample condense around the fully coordinated ligand shells in analogy to the formation of micelles by surfactant molecules that exceed their critical micellar concentration in solution. The NMR data suggests that this condensation process occurs to such an extent in the 7.5:1 CdSe sample so as to produce a multilayer ligand shell, which we will call a ligand corona here and in the following discussion. We will see that this ligand corona inhibits the ability for ligands attached to the 7.5:1 CdSe QD surfaces to undergo photoinduced detachment.

Similar to the TRIR measurements of the 3.0:1 CdSe QDs in solution (Figure 1), we examined the photoinduced ligand detachment kinetics of OA from 4.5:1, 6.0:1, and 7.5:1 CdSe QDs with increased ligand shell densities. Figure 5A represents TRIR spectra measured in the 4.5:1 CdSe QD sample in CCl_4_ following 532 nm excitation under identical conditions to those used for the 3.0:1 sample. The TRIR spectra are plotted on a logarithmic scale and exhibit qualitatively similar features to the 3.0:1 sample including the broad 1S-1P electronic transition (dotted line under the spectra), the positive-going Fano resonance features (green shading), and the free acid peak (gray shading) appearing around 1720 cm^–1^. The TRIR spectra of the 4.5:1 CdSe sample are also plotted on a linear scale in Figure S1 for reference. The same spectra appear in Figure S10 with their broad 1S-1P electronic transitions subtracted as we did for the 3.0:1 sample to highlight the free acid peak on a linear scale. The TRIR spectra of the 6.0:1 and 7.5:1 CdSe QD samples are plotted in the same ways in the figures as well.

**Figure 5:**
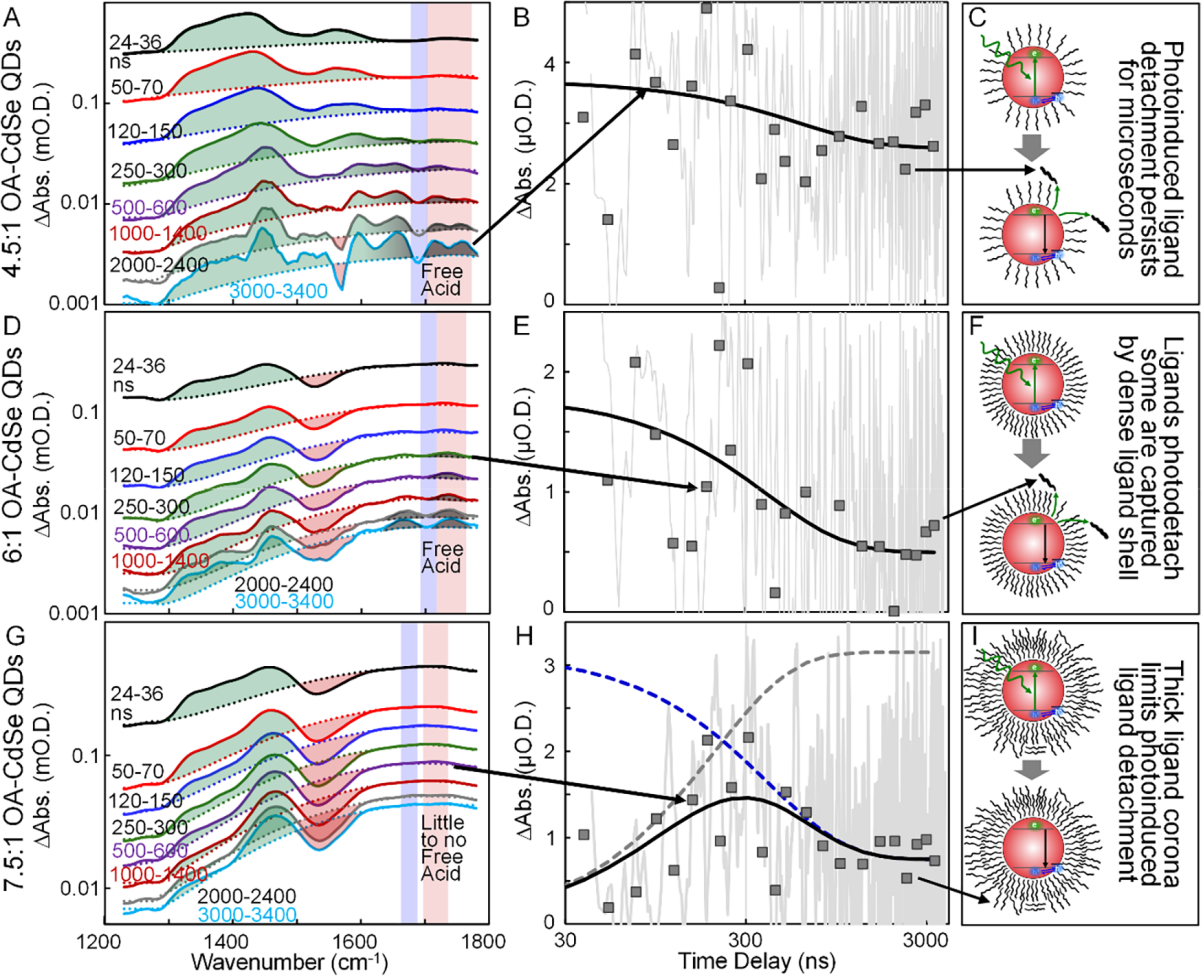
(A, D, G) Time-resolved infrared spectra at a range of time delays of solutions of 4.5:1, 6.0:1, and 7.5:1 CdSe QDs in CCl_4_ following excitation at 532 nm. The transient spectra are plotted on a logarithmic scale and time-averaged in the same manner as Figure 1C. Broad 1S-1P intraband transitions and Fano resonance features are observed in the spectra similar to the 3.0:1 CdSe QD sample. The appearance of the C = O stretch of photodetached free acid ligands (gray shading) depend on the ligand shell density as discussed in the text. The red shading highlights where kinetics of the free acid peak were measured in each sample, while the blue shading marks where the kinetics of the 1S-1P transition were determined. (B, E, H) Free acid kinetics of the 4.5:1, 6.0:1, and 7.5:1 CdSe QDs appears as the gray scattered lines and are obtained by subtraction of transient absorption kinetics traces measured at the frequency of the free acid peak versus the 1S-1P transition. The square symbols are obtained by time-averaging the primary free acid kinetics data over time intervals matched to those used to plot the transient absorption spectra. The curves through the square symbols are guides to the eye and reflect biexponential decay functions whose parameters are represented in Table S3. The time-dependence of the free acid kinetics depend sensitively on the ligand shell density and reflect the influence that interactions among ligands have on the photoinduced detachment process and on the rate of ligand recapture by the QD surfaces. (C, F, I) Cartoon illustrations of the influence that the strength of interactions among ligands have on the overall photodetachment and reattachment process at the surfaces of CdSe QDs in solution.

We verified the Fano-type nature of the green shaded resonances of the 4.5:1 CdSe QD sample by comparing the kinetics of the 1S-1P transition and the Fano resonances in Figure S11A. Similarly, we extracted the kinetics of the free acid peak represented in Figure S12A by subtracting kinetics measured at 1680 cm^–1^ of the 1S-1P transition from kinetics measured at 1720 cm^–1^ of the free acid peak. This free acid kinetics trace of the 4.5:1 sample is reproduced in Figure 5B for ease of reference. We used a similar time-averaging approach to produce the down-sampled kinetics trace indicated by the square symbols in Figure 5B. The curve through the data is a guide to the eye. The data reveal that OA ligands photodetach and become protonated within the first 10s of ns following optical excitation. Approximately 70% of these ligands remain detached throughout the microsecond time scale. The amplitude of the free acid peak of 4.0 ± 0.5 μO.D. indicates a quantum yield near unity for ligand photodetachment per absorbed photon similar to what was observed for the 3.0:1 CdSe QD sample. Figure S10B shows the prompt appearance of the free acid peak in TRIR spectra of the 4.5:1 CdSe QD sample for reference. Figure S11A reveals an average excited state lifetime of 190 ± 30 ns, similar to the 3.0:1 CdSe sample. Finally, FTIR spectra of the 4.5:1 sample measured before versus after prolonged exposure to the 532 nm excitation laser matching the conditions of the TRIR experiment (Figure S13) reveal that the ligand photodetachment is nearly reversible, similar to the 3.0:1 sample. We conclude therefore that both the 3.0:1 and the 4.5:1 CdSe QD samples exhibit similar photoinduced ligand detachment and reattachment yields and rates as illustrated in Figure 5C.

In contrast, the higher ligand density 6.0:1 and 7.5:1 CdSe QD samples exhibit distinct TRIR spectra that reflect differences in the structure and dynamics of ligands at their surfaces. These higher density ligand shell samples exhibit similar broad 1S-1P transitions in their TRIR spectra in Figure 5D,G because the sizes and optical bandgaps of their CdSe inorganic cores are matched to the lower ligand density samples (Figure S4). However, the transient vibrational features of the 6.0:1 and 7.5:1 CdSe QD samples exhibit a negative-going peak at 1550 cm^–1^ that does not appear in the samples with lower ligand density. The combination of the positive going (green shaded) and negative going (red shaded) features in the TRIR spectra of the 6.0:1 and 7.5:1 samples closely resembles the ‘hole associated’ carboxylate vibrational features observed in our prior studies of PbS and CdSe QDs in dry films.^34,35^ In those prior studies, ligands could not partition into the liquid phase because there was no solvent. This led to more fully coordinated nanocrystal surfaces in the dry films.

The similarity of the transient vibrational features of the more fully coordinated 6.0:1 and 7.5:1 CdSe QD samples to those observed in our prior dry film studies suggests that the vibrations provide information about the nature of the electronic states to which the ligands are coupled.^34,35^ To establish this, we first verified that the green- and red-shaded transient vibrational features in Figure 5D,G are indeed Fano-type resonances by comparing their decay kinetics with the 1S-1P transitions of the samples in Figure S11. The data reveal that both vibrational and electronic transitions decay synchronously as expected of Fano-type vibrational features. Furthermore, the higher ligand densities of the 6.0:1 and 7.5:1 CdSe QD samples result in fewer undercoordinated Cd atoms on their surfaces. This reduces the localized surface electron density in these samples. The reduction of this surface electron density would be expected to reduce the coupling between the ligand vibrational modes and the electronic states. This is consistent with the evolution in shape of the transient vibrational features toward absorptive Fano-type resonances because their shape depends sensitively on coupling.^59^ Finally, the loss of surface electron density would be expected to uncover surface hole density that is believed to form around undercoordinated surface Se atoms in CdSe QDs.^65^ The emergence of vibrational features similar to the ‘hole associated’ Fano-type features observed in our dry film sample measurements is consistent with these expected trends.^35^ Finally, we hypothesized that the increase of ligand density in the 6.0:1 and 7.5:1 CdSe QD samples should lead to longer excited state lifetimes in comparison to samples with lower ligand density because of the corresponding reduction of charge recombination centers. The best fit functions overlaid on the 1S-1P and Fano resonance decay kinetics (Figure S11) confirm that the 6.0:1 and 7.5:1 CdSe QD samples have longer excited state lifetimes of 540 ± 60 ns and 530 ± 30 ns, respectively. The best fit parameters of these functions appear in Table S1. As expected, this is consistent with more complete surface passivation and fewer recombination centers.

We extracted the time-dependence of the free acid peak of the 6.0:1 and 7.5:1 CdSe QD samples by subtracting kinetics traces measured around 1720 and 1680 cm^–1^ as indicated by the red and blue shaded lines in Figure 5D,G, respectively. The kinetics traces measured at these frequencies appear in Figure S12 and were subtracted following the same procedures as were used for the lower ligand density samples. The resulting free acid kinetics measured from the primary data (gray scattered lines) and the time-averaged, down-sampled free acid kinetics (square symbols) appear in Figures 5E,H for ease of inspection.

The down-sampled free acid kinetics data in Figure 5 reveal that the differences in density of the ligand shells in the 4.5:1, 6.0:1, and 7.5:1 CdSe samples cause marked changes in the photoinduced ligand detachment quantum yields and the time scales on which ligands are recaptured by the QD surfaces. The cartoons appearing in Figures 5C,5F,5I illustrate these changes for clarity. In particular, the curves through the down-sampled kinetics data reveal that both the 4.5:1 and 6.0:1 CdSe QD samples undergo prompt photoinduced ligand detachment. However, the 6.0:1 sample has a lower initial free acid peak absorption amplitude of 2.0 ± 0.5 μO.D., which implies a lower quantum yield for ligand photodetachment per absorbed photon, which we estimate to be 50% ± 15%. This lower quantum yield for ligand photodetachment is consistent with the lower localized surface electron density of the more highly coordinated 6.0:1 CdSe QD surfaces because most of their surface Cd atoms are passivated by carboxylate ligands. Importantly, in the 4.5:1 CdSe QD sample, approximately 70% of these ligands remain detached from the QD surfaces into the microsecond time scale. In contrast, the more dense ligand shells of the 6.0:1 CdSe QD sample cause stronger van der Waals interactions among ligands that enhance the probability that ligands are captured within their ligand shells. This causes about 30% of the initially photodetached ligands to remain detached into the microsecond time scale in the 6.0:1 CdSe sample.

The multilayer ligand corona that condensed around the 7.5:1 CdSe QDs led to distinct photoinduced ligand detachment kinetics. While we acknowledge the significant scatter in the down-sampled free acid kinetics in Figure 5H, the data exhibit a maximum around 300 ns time delay following optical excitation, which is distinct from the 3.0:1, 4.5:1, and 6.0:1 lower ligand density samples. The curve through the free acid kinetics represents the product of the 200 ns exponential growth function (gray dashed curve) and the 400 ns exponential decay function (blue dashed curve) that are overlaid on the 7.5:1 data. The NMR data (Figure 4) reveal that the complete passivation of the 7.5:1 CdSe QDs reduces further the density of undercoordinated Cd atoms on their surfaces. This reduces the probability that ligands will interact with surface electron density in the excited states of the QDs, which leads to slower photoinduced ligand detachment as indicated by the slower growth of the free acid peak. The multilayer ligand corona structure of the 7.5:1 CdSe QDs may also hinder movement of ligands from the QD surfaces by a type of cage effect that prevents the escape of ligands into the surrounding solvent. We believe a combination of these effects contributes to the slower photodetachment kinetics and rapid return of ligands to restore their bonding to QD surfaces. We note that the mechanism of ligand protonation described in ref 46. during exchange of weakly bound and free oleic acid molecules provides further support to our conclusions. In particular, the ligand corona around the 7.5:1 CdSe QDs would provide a bath of protons to enable rapid protonation of photodetached ligands that successfully separated from their QD surfaces.^46^ Therefore, the slower growth of the free acid peak in the 7.5:1 CdSe QDs results from slower detachment kinetics arising from less localized surface electron density and strong van der Waals interactions among the ligands.

Finally, comparison of the FTIR spectra of the various CdSe QD samples in Figure S13 that were measured before versus after prolonged exposure to 532 nm optical excitation provides insight about how ligand density influences the photodetachment and reattachment processes at QD surfaces. As mentioned earlier, both the 3.0:1 and 4.5:1 CdSe QD samples in CCl_4_ exhibit nearly complete reversibility for restoration of their ligand shells after extended exposure in which each quantum dot in the sample absorbed on average about 10^7^ photons over the course of the exposure. In contrast, the increased absorbance of the free acid peak of 0.05 and 0.03 of the 6.0:1 and 7.5:1 CdSe samples after prolonged exposure to the 532 nm excitation reveal an accumulation of approximately 2·10^18^ and 1·10^18^ OA molecules/cm^3^, respectively. This corresponds to an increase of concentration of OA ligands by 2–3 μM within experimental precision. The total dosage of absorbed pump photons after the prolonged exposure was approximately 4·10^22^ photons/cm^3^. The ratio of these values combined with the 50% quantum yield for photoinduced ligand detachment in the 6.0:1 CdSe sample indicates that 99.99% of the ligands that successfully photodetach are able to restore their bonding to the nanocrystal surfaces on the 100 μs time scale between excitation pulses.

This finding of efficient ligand photodetachment and subsequent reattachment indicates that the nanocrystal-ligand-solvent boundaries of CdSe QDs with dense ligand shells are quite dynamic. Despite strong van der Waals interactions that could impede the kinetics of ligand escape and recapture in the 6.0:1 CdSe QDs, the vast majority of ligands that photodetach and become protonated are able to restore their bonding to the nanocrystal surfaces. The data reveal that there is a limit however to how dynamic QD ligand shells can be. The smaller accumulation of free OA ligands in the solution of the 7.5:1 CdSe QDs is likely a result of a type of cage effect in which the multilayered structure of their ligand coronas impedes the diffusion of photodetached ligands from their surfaces (Figure 5H). This prevents most photodetached ligands from entering the bulk solution in this sample, which leads to the smaller accumulation of free OA ligands.

## Conclusion

We explored the influence that van der Waals interactions among ligands have on the yield of photoinduced ligand detachment and the rate of subsequent ligand return to the surfaces of OA-passivated CdSe QDs in solution using TRIR spectroscopy. We demonstrated that photoinduced ligand detachment does indeed occur from CdSe QDs in solution by monitoring the transient vibrational spectra of the photodetached ligands themselves. This was possible because the solution environment permits the OA ligands to be protonated after being photodetached from the QD surfaces, leading to distinct carboxylic acid absorptions in the TRIR spectra.

Then, we systematically varied the OA ligand density of CdSe QDs while keeping their optical, electronic, and quantum confined properties the same. We characterized the ligand shell densities using NMR spectroscopy of the vinyl protons of OA ligands with the aid of an internal standard. This approach allowed us to correlate the changes we observed in photoinduced ligand detachment kinetics of the QDs with the strength of van der Waals interactions among the ligands. Correlations of changes of the Fano-type vibrational features of ligands in the TRIR spectra with the densities of undercoordinated Cd surface atoms and the quantum yields for ligand photodetachment revealed that photoinduced ligand detachment from CdSe QDs is driven by the localization of surface electron density.

Analysis of our findings reveals that OA ligands attached to CdSe QDs with lower density ligand shells have a higher probability of escape for longer periods of time. Importantly, OA ligands on fully passivated CdSe QDs are still able to photodetach with moderate quantum yields. While the probability is greater that photodetached ligands will be captured within denser ligand shells of fully passivated QDs, the data reveal that a significant fraction of these photodetached ligands do escape and remain detached on the microsecond time scale. The vast majority of these ligands (99.99%) that successfully escape the denser ligand shells in fully passivated QDs are still able to diffuse back to the QDs and restore bonding to their surfaces on the 100 μs time scale.

Our findings reveal that the nanocrystal-ligand boundaries of colloidal nanocrystals and QDs in solution are dynamic with photoinduced changes of their porosity occurring on time scales ranging from nanoseconds (or faster) to tens of microseconds. The dynamic nature of the nanocrystal-ligand boundary emphasizes the importance of studying the surface environment of nanocrystals and QDs in solution. For example, we did not observe the protonated state of photodetached ligands in our prior studies on dry CdSe QD films, likely because of the limited proton diffusivity in the solvent-free film environment.^35^ CdSe QD surfaces in the dry film environment were also more fully coordinated because ligands could not partition between surface attached sites versus diffusing into the surrounding solvent. This led to the appearance of absorptive-type Fano resonance features in TRIR spectra of dry films similar to the spectra of fully coordinated CdSe QDs examined in this work.

While the studies in this work were conducted in an inert, N_2_ saturated solvent to prevent photocatalytic processes from complicating the ligand molecular dynamics, the dynamic picture of the nanocrystal-ligand environment revealed by these measurements suggests an intriguing possibility for future study. A natural inference of this work is that the transient increase of ligand shell porosity by ligand photodetachment might be used to improve the ability of photoexcited QDs to drive charge transfer processes while still allowing them to be fully passivated for photochemical and colloidal stability between excitation events.

## Supplementary Material



## Notes

Linear plots of TRIR spectra, analysis of Fano resonances and electronic transitions, tabulated kinetic modeling parameters, Fourier transform infrared spectra of samples before versus after excitation, absorbance and photoluminescence spectra of samples, NMR data and analysis to determine ligand surface density (PDF)
