# Supplementary material for: Dynamic Nanocrystal-Ligand Boundaries: Reversible Photoinduced Ligand Detachment from Quantum Dots in Solution
Source: J Am Chem Soc. 2026 Jan 15;148(3):3501–12. doi: 10.1021/jacs.5c19167 (PMC12856909; doi:10.1021/jacs.5c19167)
Supplement: Supplementary file 1 [file ja5c19167_si_001.pdf]

Supporting Information

Dynamic Nanocrystal-Ligand Boundaries: Reversible Photoinduced Ligand Detachment from Quantum Dots in Solution

*McKenna N. Grega‡, Jacob A. Cho‡, Robert A. Brown, and John B. Asbury**

Department of Chemistry, The Pennsylvania State University, University Park, PA 16802, USA

*jba11@psu.edu

‡ Denotes equal contribution

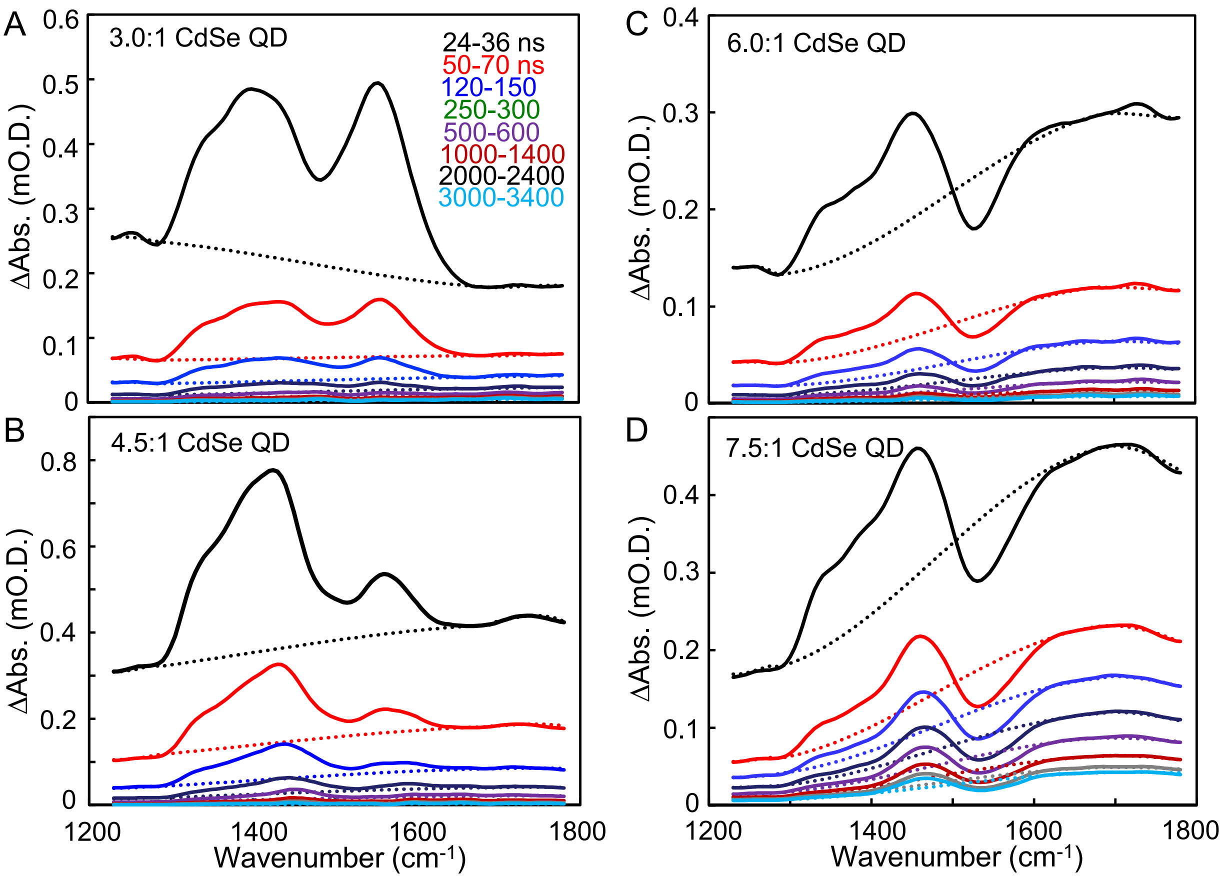

Figure S1. TRIR spectra of all four CdSe QD samples with varying ligand density are represented on a linear scale for reference. The spectra are time-averaged over the same intervals as those used in **Figure 1C**. **Panels A-D** represent the TRIR spectra for the 3.0:1, 4.5:1, 6.0:1, and 7.5:1 OA:Cd CdSe QDs, respectively as discussed in the text. The best fits of the broad 1S-1P electron absorptions appear as the dotted lines color-coded with the TRIR spectra at each time delay for each sample. For reference, the TRIR spectra and fits to the broad absorptions in panel A are identical to the spectra appearing in **Figure 1C**, except that they are plotted on a linear amplitude scale. The linear amplitude scale obscures the full dynamic range of the TRIR spectra that results from the high signal to noise ratio of the measurement. The logarithmic amplitude scale used in **Figure 1C** allows the full dynamic range of the data to be observed, which is why that representation was selected for the main text.

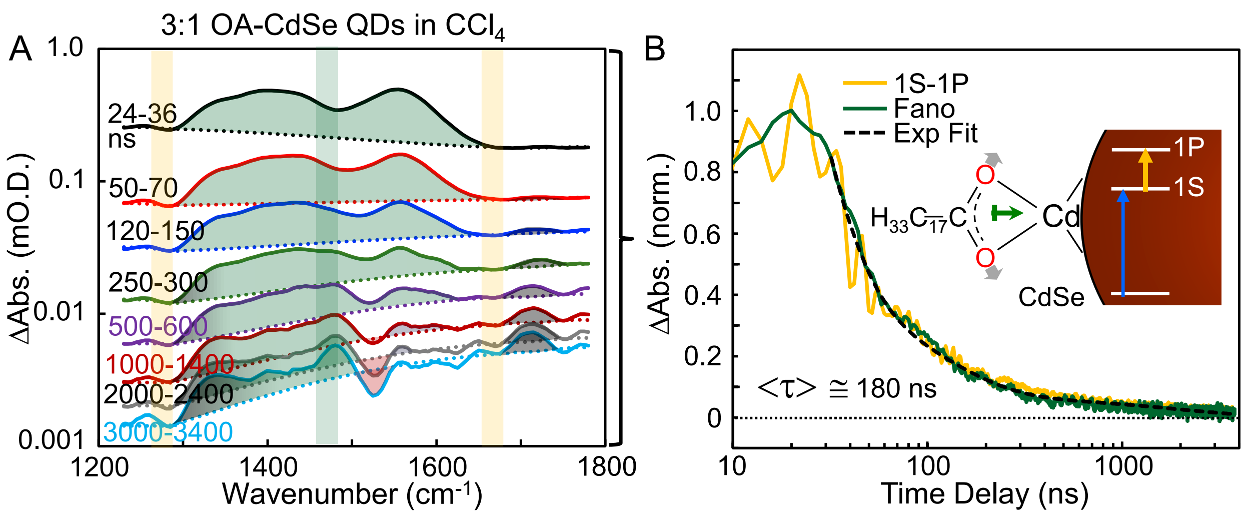

Figure S2. A. Time-resolved infrared spectra of a solution of 3.0:1 CdSe QDs in CCl_4 following excitation at 532 nm. The data are reproduced from **Figure 1C** to simplify the presentation and analysis of the data. The yellow shading highlights frequencies that were used to quantify the amplitude and time dependence of the broad 1S-1P intraband transition that is superimposed on the Fano resonance features represented by the green shaded peaks in each spectrum. The vertical green shaded band indicates the frequency at which the time dependence of the Fano resonance features was measured. **B.** The yellow kinetics trace represents the time dependence of the 1S-1P transition. The green trace depicts the time dependence of the Fano resonance amplitude, which was obtained by subtraction of the 1S-1P kinetics trace from the kinetics data measured at 1480 cm^{-1} at the center of the Fano resonances. The simultaneous decay of these features supports the assignment of the vibrational features as arising from Fano resonances that result from coupling of the molecular vibrations of ligands to the 1S-1P electronic transitions of the QDs as described in the text. The dotted line through the kinetics data represents a tri-exponential fit that was used to quantify the average decay time of the data. The best fit parameters are tabulated in **Table S1**. The inset illustrates the 1S-1P transition and the vibrational transition dipole that likely give rise to the coupling that leads to the Fano resonances.

Table S1. Best fit parameters for tri-exponential fit functions used to quantify changes of the 1S-1P and Fano resonance kinetic decays for CdSe QDs with different densities of ligand shells. The uncertainty limits of the best fit parameters are approximately ± 20%. This leads to approximately ± 20% uncertainty in the average lifetimes, which are dominated by the uncertainty of the amplitude and time constant of the slowest decay component.

	**3.0:1**	**4.5:1**	**6.0:1**	**7.5:1**
$\tau 1$ (ns)	15	20	15	15
A1	0.6	0.62	0.48	0.48
$\tau 2$ (ns)	100	120	120	120
A2	0.33	0.31	0.31	0.32
$\tau 3$ (ns)	2000	2000	2400	2400
A3	0.07	0.07	0.21	0.20
Ave. Lifetime	180	190	540	530

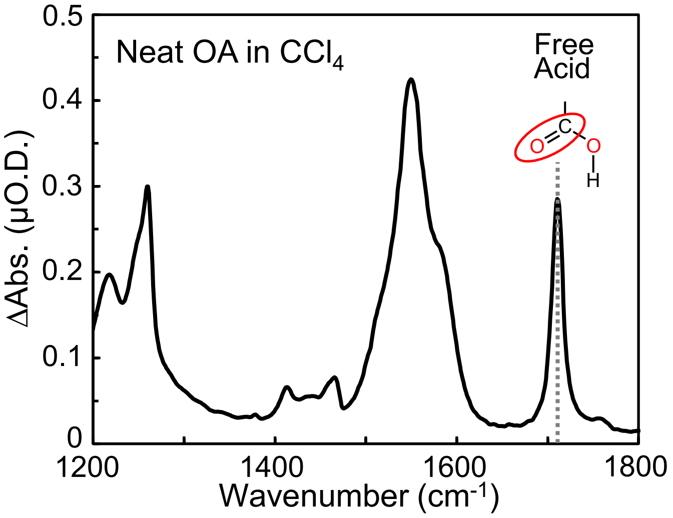

Figure S3. FTIR spectrum of a 20 mM solution of neat oleic acid in CCl_4 measured with a 150 μm optical pathlength. The free acid peak at 1720 cm^{-1} is apparent in the spectra and confirms that the 1720 cm^{-1} feature observed in the time-resolved infrared spectra of CdSe QDs in solution corresponds to the absorption of protonated oleic acid ligands. The quantitative measurement of the infrared absorption spectrum permits determination of the molar absorption coefficient of the free acid peak of ~1000 $M^{-1}cm^{-1}$ that then allows estimation of the ~70% quantum yield for photoinduced ligand detachment per absorbed photon as described in the text.

Table S2. List of the time ranges in nanoseconds over which the down-sampled kinetics data (square symbols in the Free Acid Kinetics traces) were averaged to enhance their signal to noise ratio.

Interval	Time Range	Interval	Time Range
1	24-36	13	438-498
2	38-50	14	500-600
3	52-72	15	650-750
4	74-94	16	800-900
5	96-118	17	1000-1300
6	120-150	18	1330-1630
7	152-182	19	1660-1960
8	184-214	20	2000-2300
9	216-246	21	2330-2630
10	250-300	22	2660-2960
11	310-360	23	3000-3300
12	370-430	24	3330-3630

Note S1. Description of the beam overlap factor and its use in calculation of the photoinduced ligand detachment quantum yield.

The beam overlap factor described in the main text accounts for the influence of the probe beam (1.0cm by 2.0cm spot size) being larger than the pump beam (0.8cm diameter spot) due to limitations in the ability to focus incoherent infrared radiation from the glowbar used as the IR light source in the TRIR experiment. This caused about 25% of the mid-IR probe photons to overlap the region of the sample that was excited by the pump pulse. However, all of the probe photons could impinge on the IR detector. Therefore, the mismatch in spot sizes of the pump and probe reduced the measured amplitudes of all of the transient absorption features in the TRIR spectra by reducing the observed change in transmission ΔT relative to the total transmission T of the sample (the $\Delta T/T$ ratio). We therefore used a beam overlap factor equal to four to correct for the reduced change in absorption obtained from the measured $\Delta T/T$ ratios because we sought to quantitatively interpret the change in absorption of the free acid peak to extract the concentration of photodetached ligands. This allowed us to then calculate the quantum yield for photoinduced ligand detachment of 70 ± 10%, where the uncertainty was dominated by the experimental uncertainty in the amplitude of the free acid peak of 3.0 ± 0.3 μO.D. as described in the main text.

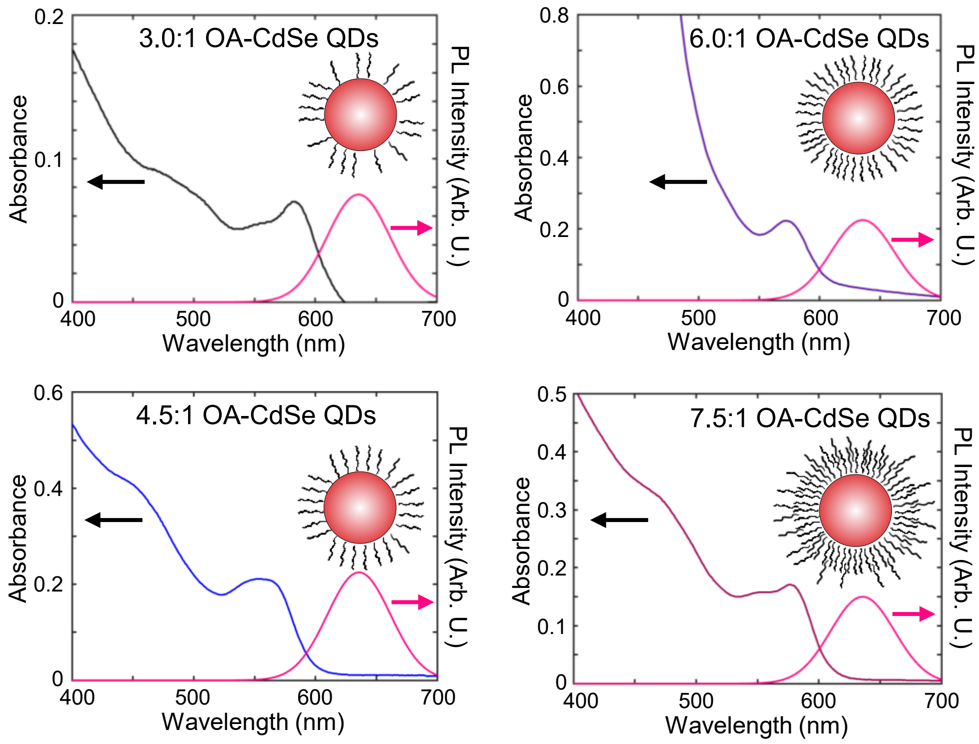

Figure S4. Visible absorption and PL spectra of CdSe QDs with various ligand shell densities examined in this work. The inset in each figure panel illustrates the ligand shell densities of each sample as determined from ^{1}H NMR analysis of the vinyl protons of oleic acid confined to the vicinity of the QDs. The ligand shell densities were changed by varying the molar ratio of oleic acid ligands versus Cd atoms in the reaction mixtures used for each synthesis as indicated in each figure. The comparison of the visible absorption and PL spectra of the samples demonstrates that they all had similar optical bandgaps, nanocrystalline sizes, and emission spectra.

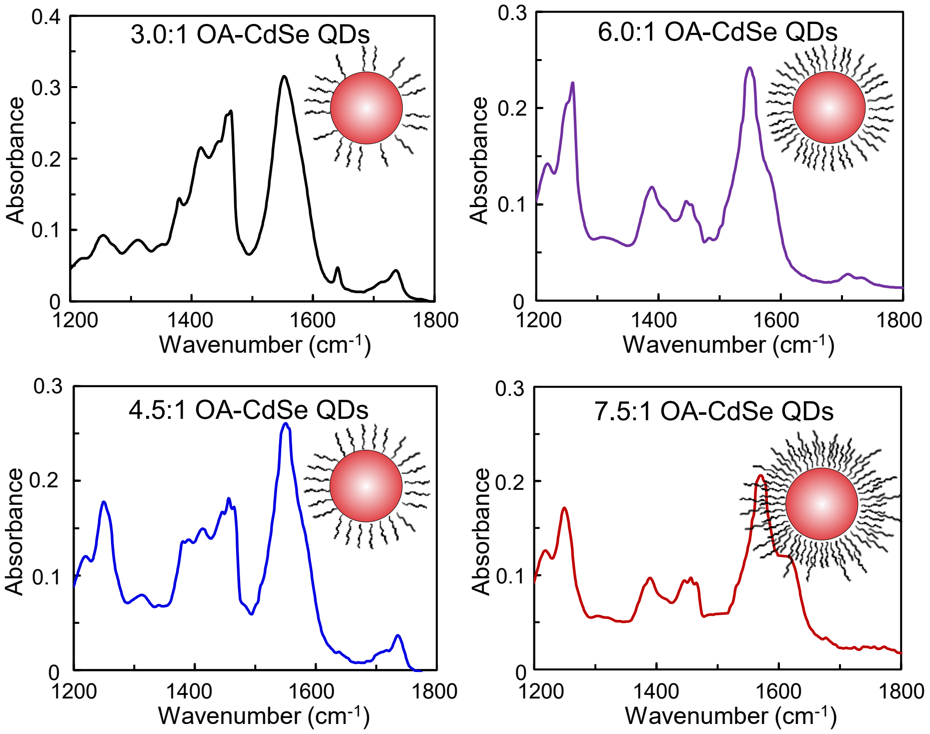

Figure S5. FTIR spectra of CdSe QDs with various ligand shell densities examined in this work. The inset in each figure panel illustrates the increase of the ligand shell densities of each sample as determined from ^{1}H NMR analysis of the vinyl protons of oleic acid confined to the vicinity of the QDs. The ligand shell densities were changed by varying the molar ratio of oleic acid ligands versus Cd atoms in the reaction mixture used for each synthesis as indicated in each figure. The comparison of the vibrational features of the ligands attached to the CdSe QD surfaces in each sample demonstrates that they all had similar ligand surface chemistry with a small amount of free oleic acid in each solution.

Note S2. Calculation of the density of ligands on QD surfaces from ^1H NMR data.

The analysis of the ligand shell density of the CdSe QDs examined in this work followed the example illustrated in the literature.[1] In particular, the concentration of oleic acid molecules confined to the vicinity of CdSe QDs was determined from the integrated intensity of the vinyl proton signal from ^1H NMR measurements of the samples in CCl_4 (**Figures S6-S9**) in comparison to the proton resonances from ferrocene in the solutions, which was introduced as an internal standard. Because the ferrocene concentration was known for each sample, the confined oleic acid concentration could also be determined by recognizing the 1:5 ratio of two vinyl protons per oleic acid molecule to the ten protons per ferrocene molecule.

The ratios of the oleic acid concentration to the CdSe QD concentration of each sample was then computed using the quantitative measurement of the excitonic absorption peak of the CdSe QDs represented in **Figure S4** for each sample.[1] We then used the approximate sizes of the CdSe QDs determined from published excitonic bandgap – nanocrystal size correlations[2] to convert the concentration of oleic acid ligands into the estimated ligand shell density for each sample. The resulting ligand shell densities are represented on the logarithmic vertical axis in **Figure 4B** of the text and are tabulated in **Table S3**.

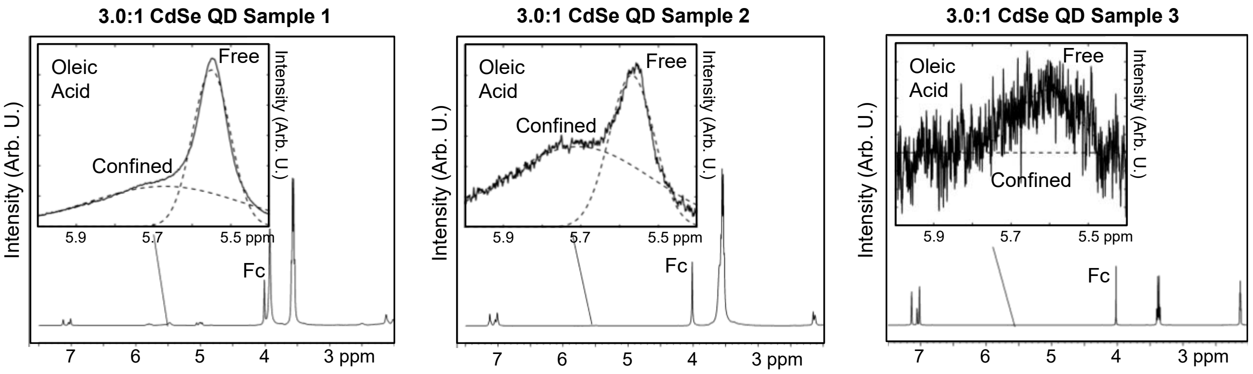

Figure S6. NMR spectra of solutions of 3.0:1 CdSe QDs in CCl_4. The spectra are represented in triplicate to show the sample-to-sample variation of the ligand surface chemistry of the nanocrystals. The inset in each panel focuses on the vinyl protons of oleic acid that exhibit distinct line shapes when they are confined to the vicinity of the CdSe QD surfaces versus when they are free to diffuse in solution. The integrated signals of the "Confined" vinyl protons were compared to the integrated signals of protons of ferrocene as the internal standard. Knowing the concentration of ferrocene introduced into each solution, the amplitude of the visible absorption of QDs in each solution, and the sizes of the nanocrystals permitted estimation of the ligand shell densities that appear in **Figure 4** of the main text. The variation of the "Free" vinyl proton signal was the result of variation of the nanocrystal purification procedures used to prepare the samples for the NMR measurements. Spectral modeling was performed to fit "Confined" and "Free" peaks (see dotted lines in each inset spectrum) so that the variation of the "Free" peak amplitude had negligible influence on the ligand shell density analysis.

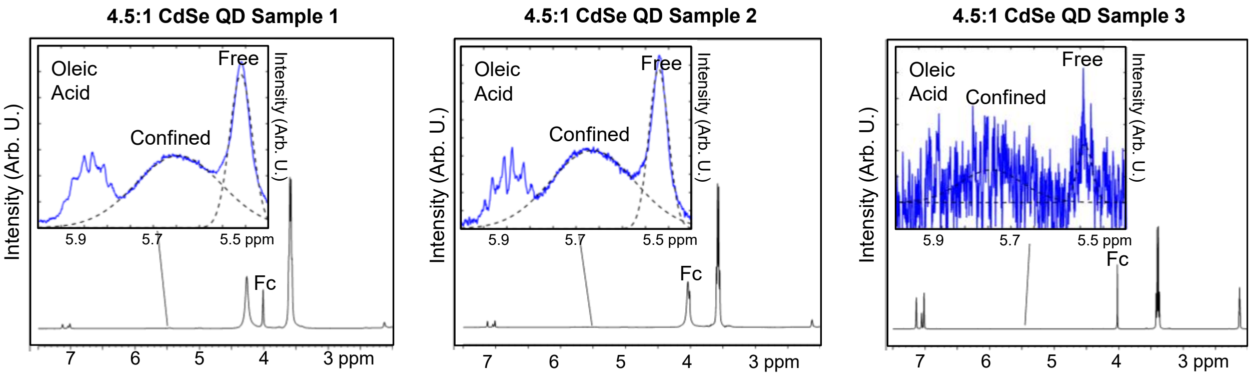

Figure S7. NMR spectra of solutions of 4.5:1 CdSe QDs in CCl_4. The spectra are represented in triplicate to show the sample-to-sample variation of the ligand surface chemistry of the nanocrystals. The inset in each panel focuses on the vinyl protons of oleic acid that exhibit distinct line shapes when they are confined to the vicinity of the CdSe QD surfaces versus when they are free to diffuse in solution. The integrated signals of the "Confined" vinyl protons were compared to the integrated signals of protons of ferrocene as the internal standard. Knowing the concentration of ferrocene introduced into each solution, the amplitude of the visible absorption of QDs in each solution, and the sizes of the nanocrystals permitted estimation of the ligand shell densities that appear in **Figure 4** of the main text. The variation of the "Free" vinyl proton signal was the result of variation of the nanocrystal purification procedures used to prepare the samples for the NMR measurements. Spectral modeling was performed to fit "Confined" and "Free" peaks (see dotted lines in each inset spectrum) so that the variation of the "Free" peak amplitude had negligible influence on the ligand shell density analysis.

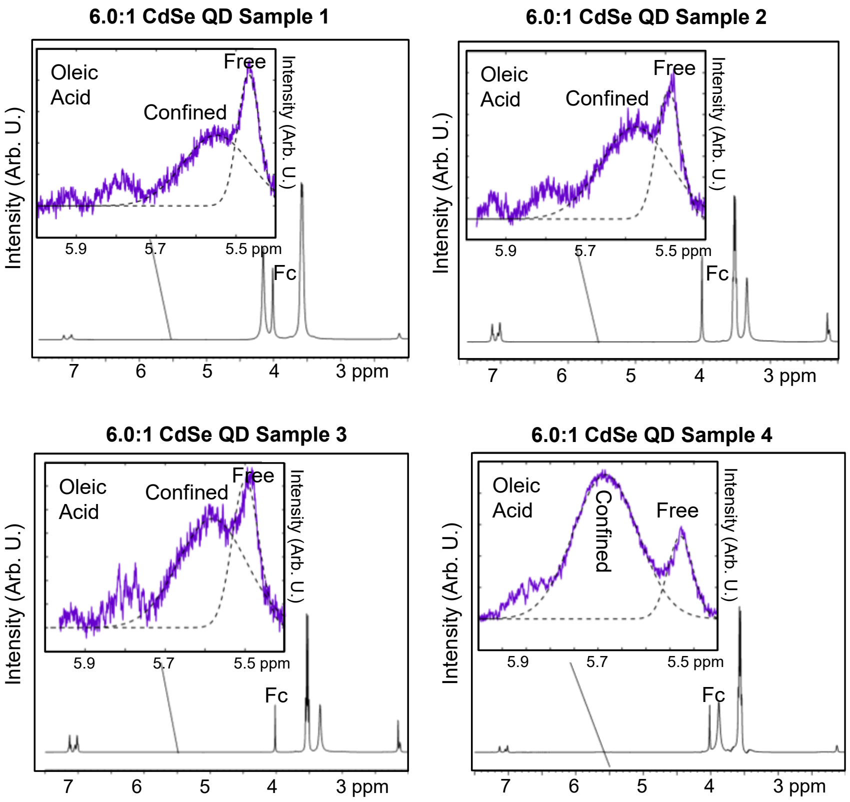

Figure S8. NMR spectra of solutions of 6.0:1 CdSe QDs in CCl_4. The spectra are represented in triplicate to show the sample-to-sample variation of the ligand surface chemistry of the nanocrystals. The inset in each panel focuses on the vinyl protons of oleic acid that exhibit distinct line shapes when they are confined to the vicinity of the CdSe QD surfaces versus when they are free to diffuse in solution. The integrated signals of the "Confined" vinyl protons were compared to the integrated signals of protons of ferrocene as the internal standard. Knowing the concentration of ferrocene introduced into each solution, the amplitude of the visible absorption of QDs in each solution, and the sizes of the nanocrystals permitted estimation of the ligand shell densities that appear in **Figure 4** of the main text. The variation of the "Free" vinyl proton signal was the result of variation of the nanocrystal purification procedures used to prepare the samples for the NMR measurements. Spectral modeling was performed to fit "Confined" and "Free" peaks (see dotted lines in each inset spectrum) so that the variation of the "Free" peak amplitude had negligible influence on the ligand shell density analysis.

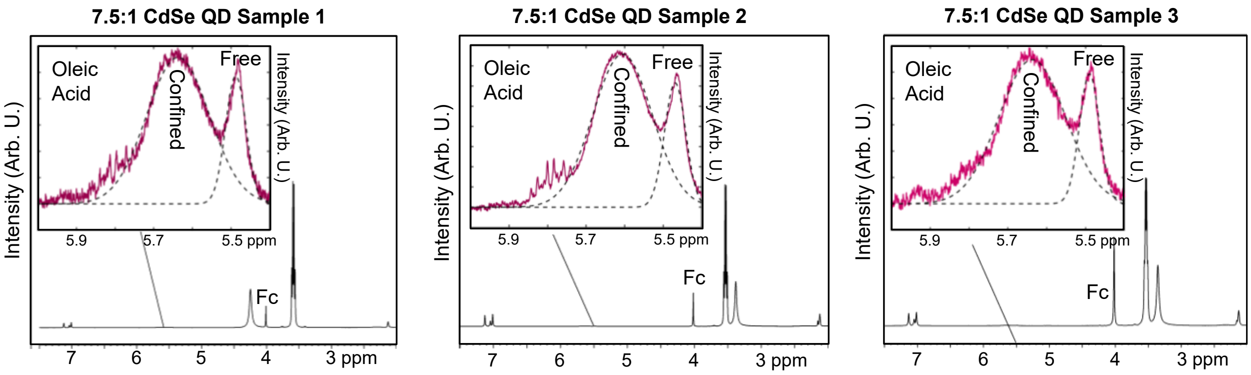

Figure S9. NMR spectra of solutions of 7.5:1 CdSe QDs in CCl_4. The spectra are represented in triplicate to show the sample-to-sample variation of the ligand surface chemistry of the nanocrystals. The inset in each panel focuses on the vinyl protons of oleic acid that exhibit distinct line shapes when they are confined to the vicinity of the CdSe QD surfaces versus when they are free to diffuse in solution. The integrated signals of the “Confined” vinyl protons were compared to the integrated signals of protons of ferrocene as the internal standard. Knowing the concentration of ferrocene introduced into each solution, the amplitude of the visible absorption of QDs in each solution, and the sizes of the nanocrystals permitted estimation of the ligand shell densities that appear in **Figure 4** of the main text. The variation of the “Free” vinyl proton signal was the result of variation of the nanocrystal purification procedures used to prepare the samples for the NMR measurements. Spectral modeling was performed to fit “Confined” and “Free” peaks (see dotted lines in each inset spectrum) so that the variation of the “Free” peak amplitude had negligible influence on the ligand shell density analysis.

Table S3. Computed ligand densities of CdSe QDs from analysis of the NMR data.

Sample	[OA]/[QD] Average	Ligand Density Average OA/nm^2
3.0:1 OA:Cd	16 +/- 2	0.35 +/- 0.1
4.5:1 OA:Cd	60 +/-32	1.2 +/- 0.5
6.0:1 OA:Cd	247 +/- 28	5.6 +/- 0.6
7.5:1 OA:Cd	2680+/- 1210	60.3 +/- 27

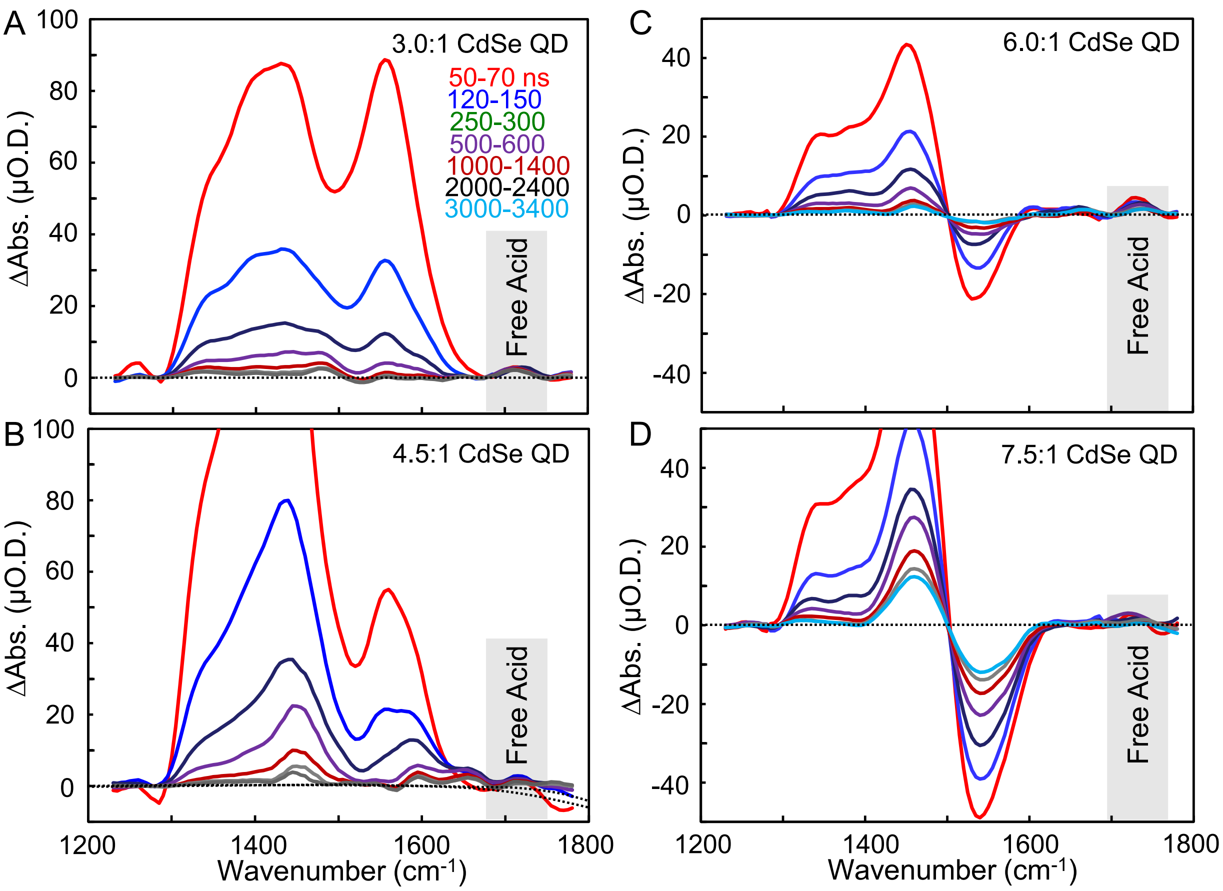

Figure S10. TRIR spectra of all four CdSe QD samples with varying ligand density are represented on a linear scale after subtraction of the broad 1S-1P electronic transitions from each spectrum. The analysis is analogous to the processing of the TRIR spectra in **Figure 2** of the 3.0:1 CdSe QDs. The spectra are time-averaged over the same intervals as those used in **Figure 5**. **Panels A-D** represent the TRIR spectra for the 3.0:1, 4.5:1, 6.0:1, and 7.5:1 OA:Cd CdSe QDs, respectively as discussed in the text. The free acid vibrational features of photodetached ligands are visible in the TRIR spectra around 1720 cm^{-1} in each sample. Subtle differences between the free acid vibrational features in these plots versus those highlighted in grey in **Figure 5** are a result of subtraction of the broad absorption offsets. The presentation of the vibrational features in **Figure 5** on a logarithmic scale does not depend on the accuracy of a mathematical description of the broad absorption offsets, which is why we chose to represent the data on a logarithmic scale in the main text.

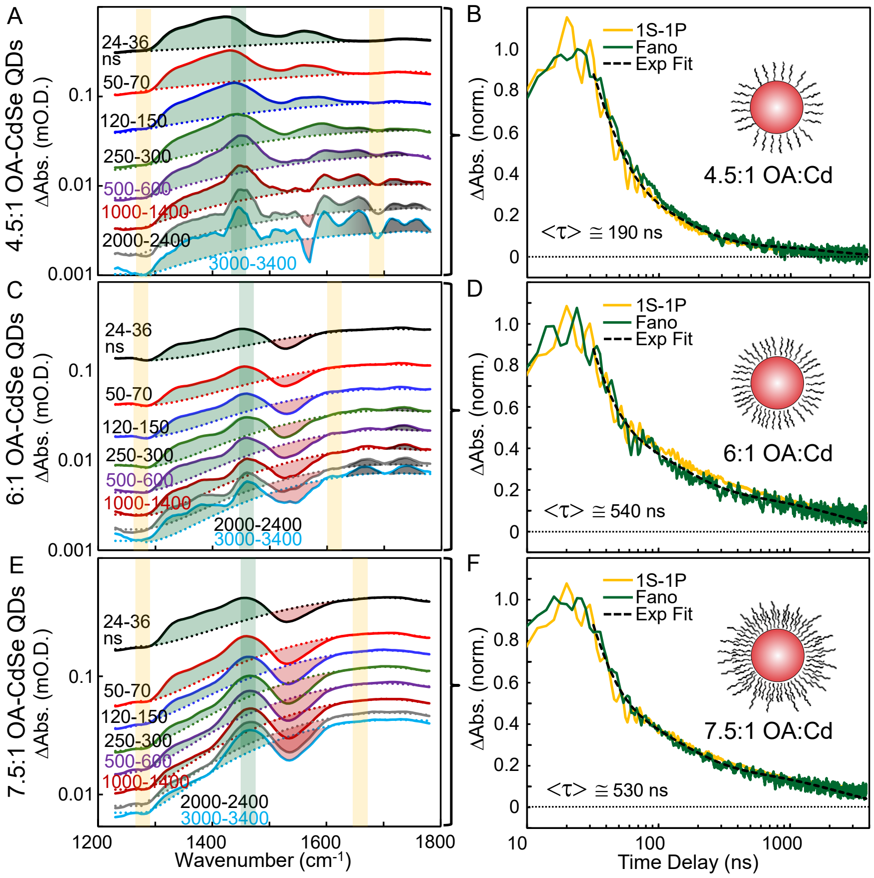

Figure S11. A, C, and **E.** Time-resolved infrared spectra of solutions of 4.5:1, 6.0:1 and 7.5:1 CdSe QDs in CCl_4 following excitation at 532 nm. The data are reproduced from **Figure 5** to simplify the presentation and analysis of the data. The yellow shading highlights frequencies that were used to quantify the amplitude and time dependence of the broad 1S-1P intraband transition in each sample that is superimposed on the Fano resonance features represented by the green shaded peaks. The vertical green shaded band indicates the frequency at which the time dependence of the Fano resonance features was measured. **B, D,** and **F.** The yellow kinetics traces represent the time dependence of the 1S-1P transitions of each sample. The green traces depict the time dependence of the Fano resonance amplitudes, which are obtained by subtraction of the 1S-1P kinetics trace from the kinetics data measured at 1480 cm^{-1} at the center of the Fano resonances. The simultaneous decay of these features in all samples supports the assignment of the vibrational features as arising from Fano resonances that occur from coupling of the molecular vibrations of ligands to the 1S-1P electronic transitions of the QDs. The dotted lines through the kinetics data represent tri-exponential fits that were used to quantify the average decay times of the data. The best fit parameters are tabulated in **Table S1**.

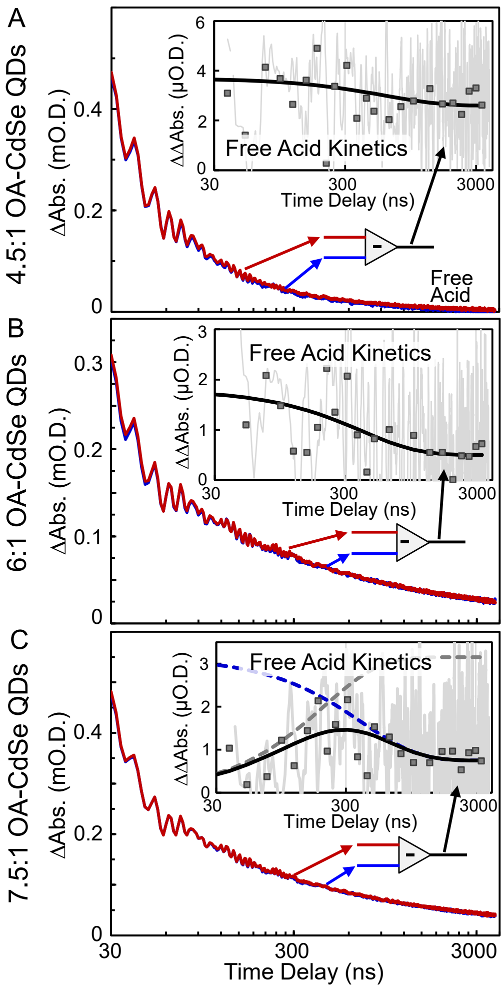

Figure S12. Transient absorption kinetics traces measured at the frequency of the free acid peaks (red traces) and 1S-1P transitions (blue traces) obtained from the time-resolved infrared spectra in **Figure 5** of the 4.5:1, 6.0:1, 7.5:1 CdSe QD samples. The difference of these traces isolates the time dependence of the free acid peaks and are represented as the grey data in the insets. The primary free acid kinetics data were time averaged over intervals represented by the square symbols to highlight the prompt formation and persistent lifetime of the photodetached ligands in the solutions.

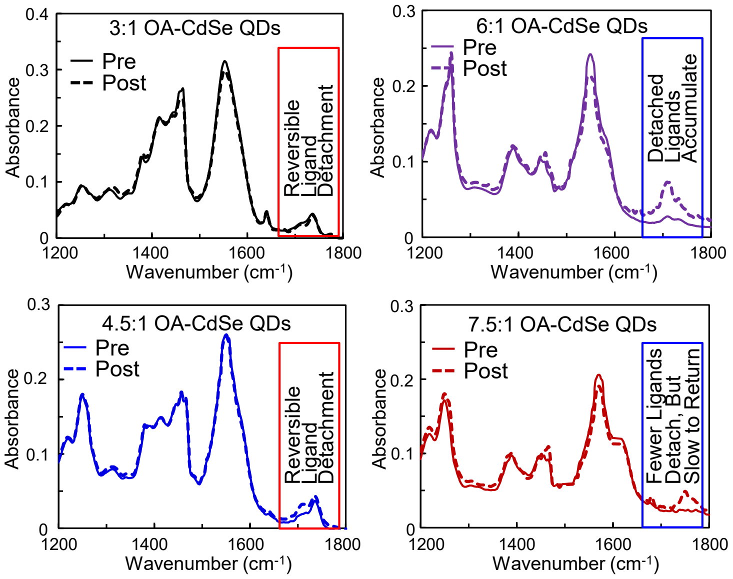

Figure S13. Comparison of FTIR spectra of solutions of 3.0:1, 4.5:1, 6.0:1, and 7.5:1 CdSe QDs in CCl_4 that were measured before versus after exposure of the sample to 20 μJ/cm^2 532 nm excitation with 5 kHz repetition rate over a four-hour period. Under these conditions, each QD in the solution absorbed ~10^7 photons on average. The samples with lower ligand density exhibit complete reversibility of the photoinduced ligand detachment within experimental precision. However, the 6.0:1 and 7.5:1 samples with more dense ligand shells show evidence of increased free ligand concentration after the laser exposure. This is likely the result of the lower density of available surface sites in these more dense ligand shells that frustrate the bimolecular collisions that lead to ligand recapture.

References

(1) Roberge, A.; Dunlap, J. H.; Ahmed, F.; Greytak, A. B., Size-Dependent Pbs Quantum Dot Surface Chemistry Investigated Via Gel Permeation Chromatography. *Chem. Mater.* **2020**, *32*, 6588-6594.

(2) Yu, W. W.; Qu, L.; Guo, W.; Peng, X., Experimental Determination of the Extinction Coefficient of CdTe, CdSe, and CdS Nanocrystals. *Chem. Mater.* **2003**, *15*, 2854-2860.
